# Successful Unilateral Ventro-Oral (Vo) Thalamotomy for Peripheral Post-traumatic Dystonia With Complex Regional Pain Syndrome: A Case Report

**DOI:** 10.7759/cureus.83536

**Published:** 2025-05-05

**Authors:** Yoji Kuramoto, Takaomi Taira, Shoichiro Tsuji, Takanori Kubo, Shinichi Yoshimura

**Affiliations:** 1 Neurosurgery, Hyogo Medical University, Nishinomiya, JPN; 2 Functional Neurosurgery, Kumagaya General Hospital, Kumagaya, JPN

**Keywords:** fixed dystonia, functional dystonia, limb dystonia, stereotactic brain lesioning, ventro-oral thalamotomy

## Abstract

Peripheral post-traumatic dystonia (PPD) and complex regional pain syndrome (CRPS) are both challenging conditions often stemming from trauma and, in some cases, coexisting. This complexity complicates diagnosis and treatment approaches. A case involving a woman in her 40s highlighted this issue - after a hand injury, she suffered from deformities and pain. Standard treatments were ineffective, but ventro-oral (Vo) thalamotomy brought significant symptom relief. Despite this success, treating PPD, especially when mixed with CRPS, remains difficult, and tailored approaches are crucial. Further research is essential to better understand and manage these conditions.

## Introduction

Peripheral post-traumatic dystonia (PPD) is caused by trauma to non-central peripheral nerves, where the symptoms are acute to subacute, and the limb position abnormality is often fixed regardless of movement. It can be classified as functional or fixed dystonia [1]. Complex regional pain syndrome (CRPS) is one of the neuropathic pain disorders caused by neurodegenerative diseases, trauma, and infection. CRPS is often challenging to treat because of the intense and persistent pain it causes [2,3]. Involuntary movements are associated with CRPS in 25-30% of cases [4,5]. When both PPD and CRPS are present, the symptoms may be different, making diagnosis and treatment difficult.

We report on a patient who suffered from PPD and CRPS for approximately 20 years following a hand injury and underwent thalamic ventrooral nucleus (Vo)-thalamotomy to improve symptoms of fixed dystonia and increasing activities of daily living (ADL).

## Case presentation

**Figure 1:**
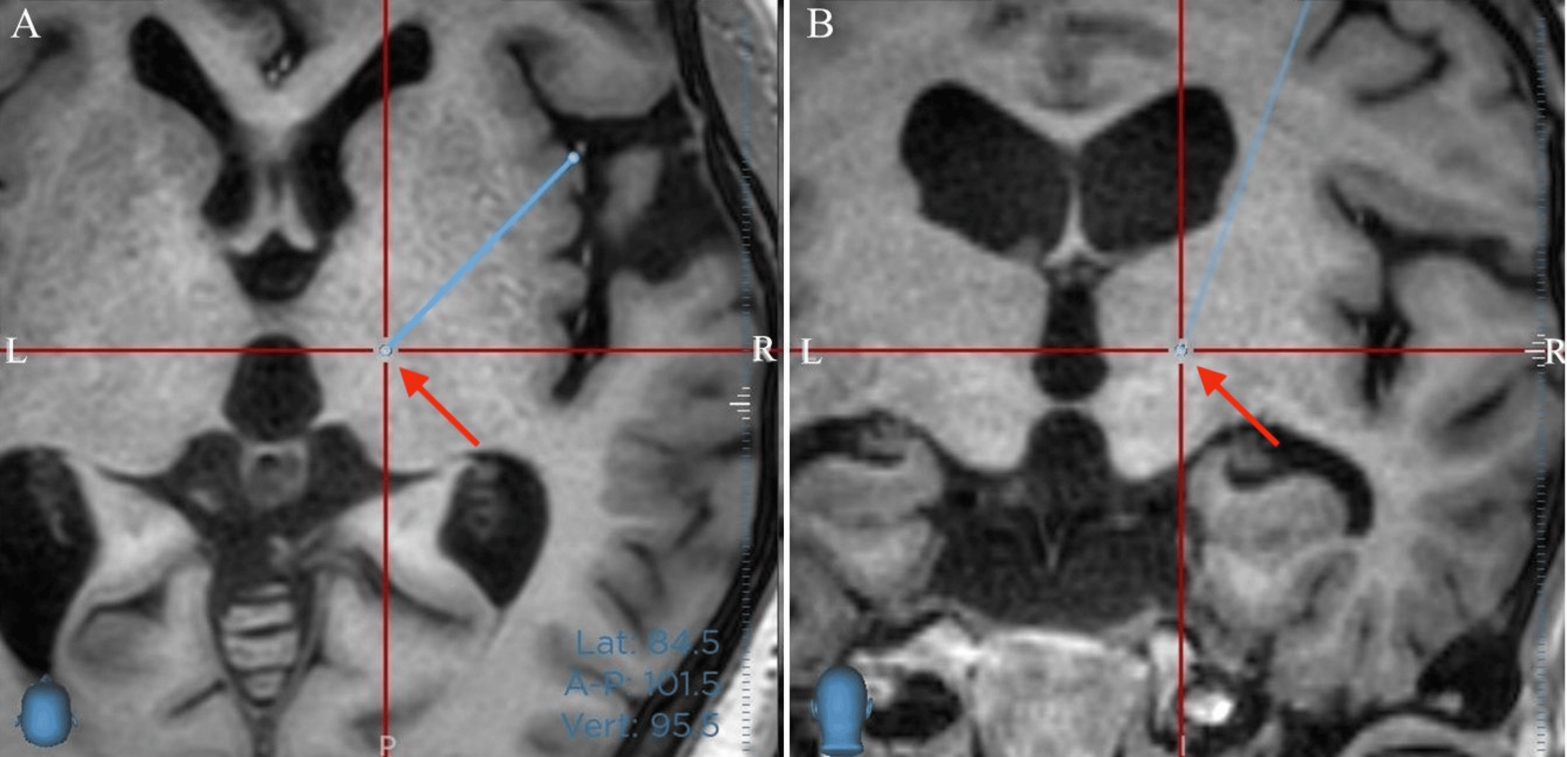
Preoperative target is in the red cross line center. A: Axial, B: Coronal sections on T1WI. Unlike a regular MRI, the screen's right side corresponds to the patient's right side. The light blue line points to the trajectory and the red arrow points to the target.

A woman in her 40s was referred with a chief complaint of abnormal limb position, paresthesia, and hypersensitivity in her left limbs. Twenty years ago, she injured her left hand with scissors and developed tingling and severe pain in that hand. One month later, abnormal limb posture appeared in her left hand. Over time, the limb's abnormal position and the pain's extent gradually spread to other body parts. She received an intrathecal baclofen pump (ITB) implantation at another hospital but was not satisfied with the results, so it was removed. The botulinum toxin injections were inconsistent and did not improve muscle tone or correct limb position abnormalities. For pain management, she was prescribed valproic acid and gabapentin due to the adverse effects of carbamazepine and pregabalin; however, she reported no relief from the pain. Upon presentation, her left upper limb exhibited a claw hand deformity, and her left foot showed a rotated ankle joint with restricted motion (Video 1). The Burke-Fahn-Marsden Dystonia Rating Scale (BFMDRS), which is used to assess the severity and disability of dystonia, was 53. However, tendon reflexes in both limbs were not increased. However, opposite abnormal limb positions appeared when the pain increased. Based on these findings, a diagnosis of PPD was made, in which spasticity was not a contracture but a limb position abnormality caused by persistent muscle tension. As dystonia was predominantly in the left limb, a right Vo thalamotomy was aimed at improving symptoms of involuntary movements. The surgery was performed with a Lekcell frame and a Stealth Station V8 (Medtronic, Minneapolis, MN, USA). Fine-tuned with MRI, the final target was right lateral: 14.85 mm, posterior: 2.87 mm, and superior: 3.01 mm from the midcommissural point (MCP) (Figure 1).

**Video 1:** Pre-operative movie Muscle tonus and abnormal limb position affect the distal left upper and lower limbs, especially when walking.

**Figure 2:**
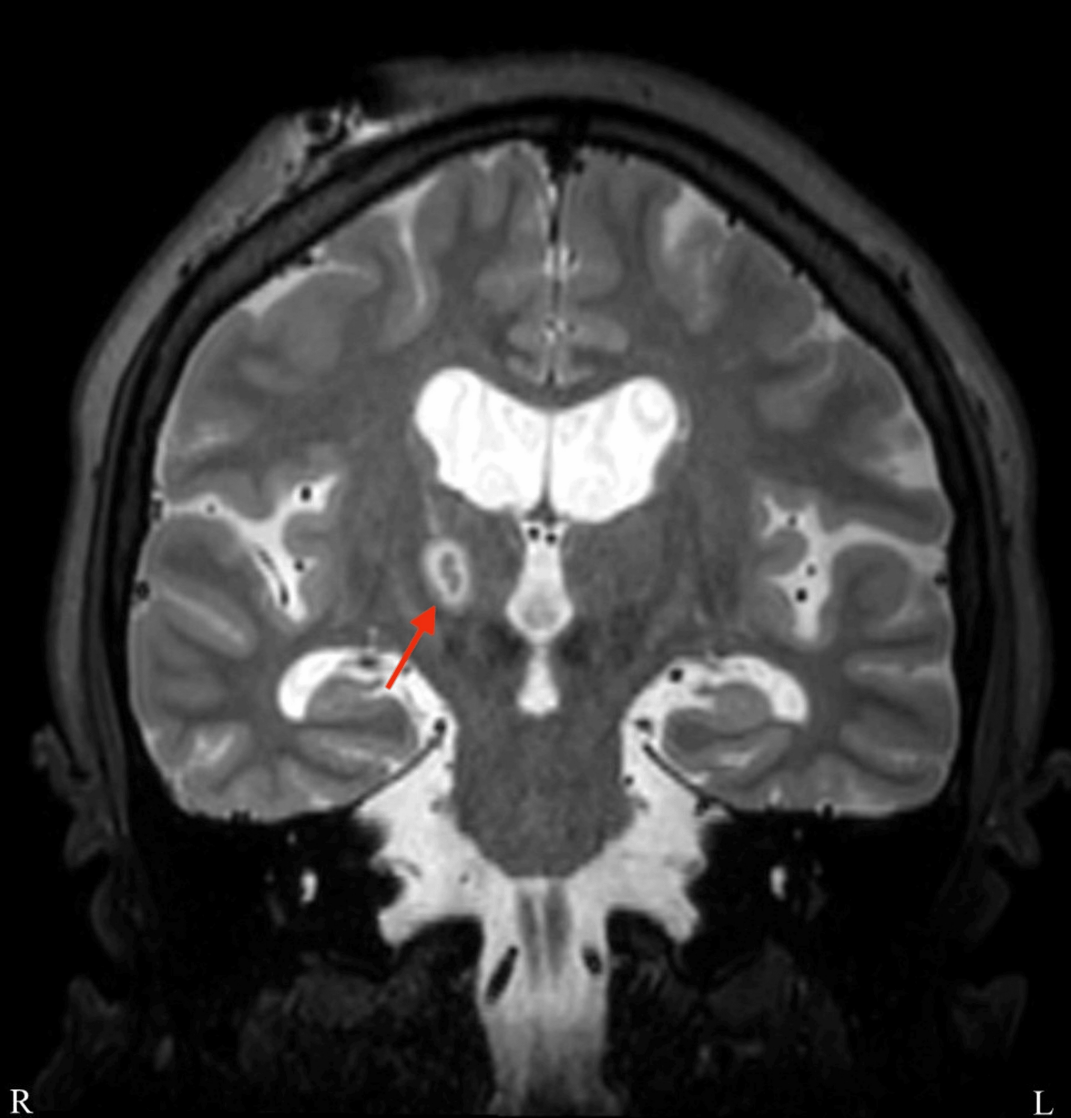
Postoperative coronal T2 weight image. As in a normal MRI, the left side of the screen is the patient's right. The coagulation area is almost identical to the previous target. The red arrow points to the lesioning area.

The operation was performed with local anesthesia and without intravenous sedation. Following insertion of the lesioning needle into the Vo nucleus, electrical test stimulation was performed at 130 Hz, 100 us, 3 mA. During the stimulation, no side effects were observed. After lesioning at a depth of 0 and -3 mm at 70℃ for 40 seconds, the patient's leg symptoms disappeared, and she could move it smoothly. Additional lesioning of the lateral posterior Vo-Vim border, the most effective point for limb dystonia, further improved the hand symptoms. Post-operative MR confirmed the appropriate lesioning (Figure 2).

After surgery, the patient's abnormal limb position disappeared when standing and walking; the BFMDRS scale score was 1, and three months later, the BFMDRS was 5, with sustained improvement (Video 2).

**Video 2:** Intraoperative movie An electrode was inserted to the right Vo nucleus, and coagulation was performed after electrical test stimulation. There were no apparent adverse reactions, and the patient showed improvement in symptoms, especially in the left leg during the operation. Symptomatic improvement has been maintained postoperatively.

## Discussion

The classification of trauma-induced dystonia (posttraumatic dystonia, PD) encompasses two distinct forms: central and peripheral dystonia, the former of which is characterized by damage to the central nervous system, and the latter by peripheral injury, like this case. PD is classified as secondary dystonia and is often challenging to treat; the first documented report of PPD was published in 1888 by Gowers, who reported the occurrence of abnormal involuntary movements following peripheral local trauma to the neck and thumb.

Both PPD and functional dystonia (fixed dystonia) are often classified similarly [6]. The presence of CRPS may be attributed to the alleged trauma. The main problem in this case was that her limbs were in an abnormal limb posture, causing difficulty in walking and daily living. The patient and her family agreed that surgery was not for pain relief but to release the abnormal limb posture and muscle tonus.

Fixed dystonia (or PPD) occurs in about 25% of patients with CRPS, which is usually triggered by a limb injury. CRPS is characterized by persistent pain and autonomic and trophic features [4,6]. The present case demonstrates a marked similarity to the Wernicke-Mann limb position frequently observed in patients suffering from post-stroke hemiplegia. The hand exhibits a distinctive claw hand, a characteristic that has also been documented in the context of functional dystonia [7].

ITB implantation would have been a therapeutic intervention if spasticity contributed to the symptoms. It may also be performed when stereotactic brain surgery is ineffective. However, this treatment had already been initiated and was ineffective. We hypothesized that the abnormal sensation was challenging to treat but that the dystonia was amenable to treatment. The standard treatment for PPD comprises botulinum toxin injection and medication; however, the efficacy of these treatments is low, with a success rate of approximately 20%. Some reports suggest that rehabilitation treatment should be provided, and surgical treatment should be avoided [8]. There are cases in which surgical treatment has been dramatically effective, as in the present report, and it is not advisable to assume a psychogenic origin for all cases [8]. In this case, the muscle tonus and abnormal limb position were hypothesized to be attributable to the PPD, not psychogenic. Consequently, Vo thalamotomy was selected as renowned for its efficacy in treating focal limb dystonia [9]. However, there are reports of the ineffectiveness of deep brain stimulation (DBS) with the globus pallidus internus (GPi) and thalamus to PPD, and its limitations need to be fully explained preoperatively [10]. Transcranial magnetic stimulation is one of the new approaches to dystonia [11]. This new approach was already adapted to depression and obsessive-compulsive disorder, such as psychiatric disorders [12]. But the effectiveness of this approach was unclear for PPD. Psychiatric disorders complicate PPD, so more effective treatment may be possible, but it is a subject for future investigation.

This report constitutes a single case report that aims to ascertain the effectiveness of stereotactic brain surgery for secondary dystonia, such as PPD. Therefore, future work should be conducted to accumulate many similar cases and clarify the efficacy rates and complications.

## Conclusions

This case demonstrates the need for careful observation to identify potentially ameliorative symptoms of PPD and improved ADL. The case also indicates that stereotactic brain surgery is effective. Therefore, many similar cases in the future are necessary to clarify the efficacy rates and complications.
